# Changes in adolescents’ planned hospital care during the COVID-19 pandemic: analysis of linked administrative data

**DOI:** 10.1136/archdischild-2021-323616

**Published:** 2022-05-16

**Authors:** Louise Mc Grath-Lone, David Etoori, Ruth Gilbert, Katie L Harron, Jenny Woodman, Ruth Blackburn

**Affiliations:** 1 Institute of Health Informatics, University College London, London, UK; 2 UCL Great Ormond Street Institute of Child Health, London, UK; 3 Social Research Institute, University College London, London, UK

**Keywords:** Covid-19, Adolescent Health, Child Health Services, Social work

## Abstract

**Objective:**

To describe changes in planned hospital care during the pandemic for vulnerable adolescents receiving children’s social care (CSC) services or special educational needs (SEN) support, relative to their peers.

**Design:**

Observational cohort in the Education and Child Health Insights from Linked Data database (linked de-identified administrative health, education and social care records of all children in England).

**Study population:**

All secondary school pupils in years 7–11 in academic year 2019/2020 (N=3 030 235).

**Main exposure:**

Receiving SEN support or CSC services.

**Main outcomes:**

Changes in outpatient attendances and planned hospital admissions during the first 9 months of the pandemic (23 March–31 December 2020), estimated by comparing predicted with observed numbers and rates per 1000 child-years.

**Results:**

A fifth of pupils (20.5%) received some form of statutory support: 14.2% received SEN support only, 3.6% received CSC services only and 2.7% received both. Decreases in planned hospital care were greater for these vulnerable adolescents than their peers: −290 vs −225 per 1000 child-years for outpatient attendances and −36 vs −16 per 1000 child-years for planned admissions. Overall, 21% of adolescents who were vulnerable disproportionately bore 25% of the decrease in outpatient attendances and 37% of the decrease in planned hospital admissions. Vulnerable adolescents were less likely than their peers to have face-to-face outpatient care.

**Conclusion:**

These findings indicate that socially vulnerable groups of children have high health needs, which may need to be prioritised to ensure equitable provision, including for catch-up of planned care postpandemic.

## Abstract (precis)

This paper describes changes in planned hospital care for adolescents during the pandemic, highlighting the disproportionate impact on those receiving children’s social care services or special educational needs support.

**What is already known on this topic**

- Planned hospital care (outpatient attendances and planned hospital admissions) was disrupted during the COVID-19 pandemic.
- In England, children experienced greater relative decreases in planned hospital care than adults, but we lack evidence on which groups were most impacted.
- Children receiving special educational needs (SEN) support or children’s social care (CSC) services experience poorer health, education and social care outcomes than their peers and may have been more vulnerable to the indirect effects of the pandemic, such as disruptions to healthcare access.

**What this study adds**

- Before the pandemic, adolescents receiving SEN support or CSC services had higher rates of planned hospital care than their peers.
- During the pandemic, there were large decreases in planned care for adolescents overall, which disproportionately affected 21% receiving SEN support or CSC services, who bore 25% of the decrease in outpatient attendances and 37% of the decrease in planned hospital admissions.
- Vulnerable adolescents were less likely than their peers to have face-to-face outpatient care during the pandemic.

**How this study might affect research, practice or policy**

- This study shows that children receiving statutory services have greater use of planned hospital care than their peers and were more affected by disruptions during the pandemic.
- These findings provide empirical evidence to inform policy prioritisation of vulnerable groups of children who have high health needs to ensure equitable provision of care that is accessible and appropriate, including for catch-up of planned care postpandemic.
- Further research using linked health, education and social care data is needed to understand the potential consequences of delayed or foregone planned hospital care (such as diagnostic assessments or treatments) for young people.

## Introduction

Compared with adults, the direct effects of COVID-19 on young people, in terms of serious infections and deaths, have been relatively low.^1 2^ However, young people have experienced considerable indirect effects of the pandemic through disruptions to health and other services, including much greater relative decreases in planned hospital admissions than adults.^3^ Among adults, disruptions to healthcare during the pandemic have not been borne equally,^4 5^ and it is likely that certain groups of young people have also been disproportionately affected.

In the Childhood Vulnerability and COVID-19 framework developed by Public Health England,^6^ children receiving statutory support/services were considered to be more vulnerable to the indirect effects of the pandemic due to family and social circumstances. Based on this framework, we hypothesised that adolescents receiving children’s social care (CSC) services or special educational needs (SEN) support were likely to have been more affected by the large reductions in planned hospital care during the pandemic as they have higher rates of chronic health conditions than their peers.^7 8^

This analysis aimed to describe changes in planned hospital care during the pandemic among vulnerable adolescents receiving CSC services and/or SEN support. We focused on planned hospital care (ie, outpatient appointments and planned hospital admissions) because it is used to investigate, monitor, manage and treat young people’s health needs. Therefore, decreases that occurred during the pandemic may indicate deferred or unmet health needs.

## Methods

### Data source and study population

We analysed the Education and Child Health Insights from Linked Data (ECHILD) database,^9^ a whole population data set that links de-identified administrative health, education and social care records of all children in England. The ECHILD database contains hospital records of all National Health Service (NHS) patients in England, as captured by Hospital Episodes Statistics (HES). It also includes information about the characteristics of pupils in all state-maintained schools and other educational settings (such as pupil referral units and alternative provision) and about CSC referrals, assessments and interventions, as captured by the National Pupil Database (NPD).

We included all secondary school pupils in years 7–11 in academic year 2019/2020 (typically aged 11–16 years). Pupils enrolled in private schools (approximately 7% each year^10^) or home-schooled (<1% each year prepandemic^11^) could not be included as NPD does not collect information for these groups.

### Exposure

We identified pupils receiving SEN support or CSC services before the pandemic began based on the most recent education and social care information recorded in the ECHILD database (2019/2020 for SEN and 2018/2019 for CSC; online supplemental figure 1). The NPD is a statutory data collection used to produce national statistics about SEN support and CSC services involvement^12 13^; therefore, it is a reliable source of information about the study exposure.

10.1136/archdischild-2021-323616.supp1 Supplementary data

To align with the Childhood Vulnerability and COVID-19 framework, we described outcomes for all adolescents receiving SEN support and/or CSC services. We also chose to describe outcomes by type of statutory services (SEN support only, CSC services only, both SEN and CSC services) as these interventions are used for different purposes and it is likely that those who receive them differ in terms of their background characteristics and health needs.

### Outcomes and statistical analyses

The primary outcome for this analysis was the decrease in planned hospital care (outpatient attendances and planned hospital admissions). HES data are collected for the purpose of reimbursing hospitals for the care they have delivered and, as the vast majority of hospital care in England is delivered by the NHS,^14^ it is likely that it is an accurate source of outcome data.

It was only possible to look at changes to planned hospital care during the first 9 months of the pandemic (23 March–31 December 2020) as these were the latest HES data in the ECHILD database at the time of the analysis.

First, we calculated the rates of planned hospital care per 1000 child-years in 2015–2019. From this prepandemic baseline, we predicted the expected rates in 2020 had the pandemic not happened, assuming any observed time trends between 2015 and 2019 would have continued, using Poisson models that included a linear effect of time year stratified by type of statutory support or services received. We then calculated the difference between the expected and the observed rates for each group. Based on other studies of hospital activity among children during 2020,^3^ we expected greater relative decreases in planned hospital admissions than outpatient attendances.

We also looked at the mode (inperson vs tele/virtual) of scheduled outpatient appointments (to examine differences in the type of appointments offered by hospitals) and outpatient attendances (to examine differences in the type of appointments young people chose to attend).

## Results

### Study population

Table 1 presents the key characteristics of the young people included in this analysis. Of the 3 030 235 pupils in school years 7–11 in 2019/2020, a fifth (621 137, 20.5%) were receiving statutory support/services: 14.2% SEN support only, 3.6% CSC services only and 2.7% both.

**Table 1:** Characteristics of pupils in school years 7–11 in 2019/2020, by type of statutory support or service

Type of statutory support or services	Overall (N=3 030 235)		Not supported or receiving services		Supported or receiving services		SEN only		CSC only		Both SEN and CSC	
			n	%	n	%	n	%	n	%	n	%
			2 409 098	79.5	621 137	20.5	428 964	14.2	110 390	3.6	81 783	2.7
	n	%	n	%	n	%	n	%	n	%	n	%
School year group												
Year 7	644 073	21.3	504 108	20.9	139 965	22.5	100 976	23.5	22 453	20.3	16 536	20.2
Year 8	620 524	20.5	492 987	20.5	127 537	20.5	89 995	21.0	21 771	19.7	15 771	19.3
Year 9	601 119	19.8	480 987	20.0	120 132	19.3	81 936	19.1	22 230	20.1	15 966	19.5
Year 10	590 050	19.5	472 898	19.6	117 152	18.9	78 915	18.4	21 904	19.8	16 333	20.0
Year 11	574 469	19.0	458 118	19.0	116 351	18.7	77 142	18.0	22 032	20.0	17 177	21.0
Gender												
Male	1 553 539	51.3	1 172 080	48.7	381 459	61.4	280 761	65.5	48 075	43.6	52 623	64.3
Female	1 476 236	48.7	1 236 679	51.3	239 557	38.6	148 131	34.5	62 293	56.4	29 133	35.6
Ethnic group												
Asian	327 228	10.8	280 719	11.7	46 509	7.5	32 495	7.6	9602	8.7	4412	5.4
Black	176 468	5.8	140 624	5.8	35 844	5.8	22 886	5.3	8010	7.3	4948	6.1
Mixed	172 723	5.7	134 375	5.6	38 348	6.2	23 510	5.5	8952	8.1	5886	7.2
White	2 214 274	73.1	1 743 155	72.4	471 119	75.8	331 211	77.2	77 672	70.4	62 236	76.1
Other	68 071	2.2	57 909	2.4	10 162	1.6	7146	1.7	2027	1.8	989	1.2
Unknown	71 471	2.4	52 316	2.2	19 155	3.1	11 716	2.7	4127	3.7	3312	4.0
Free school meal eligibility												
No	2 491 209	82.2	2 081 712	86.4	409 497	65.9	312 703	72.9	56 521	51.2	40 273	49.2
Yes	539 026	17.8	327 386	13.6	211 640	34.1	116 261	27.1	53 869	48.8	41 510	50.8

CSC, children’s social care services; SEN, special educational needs support.

### Planned hospital care before the pandemic

**Figure 1:**
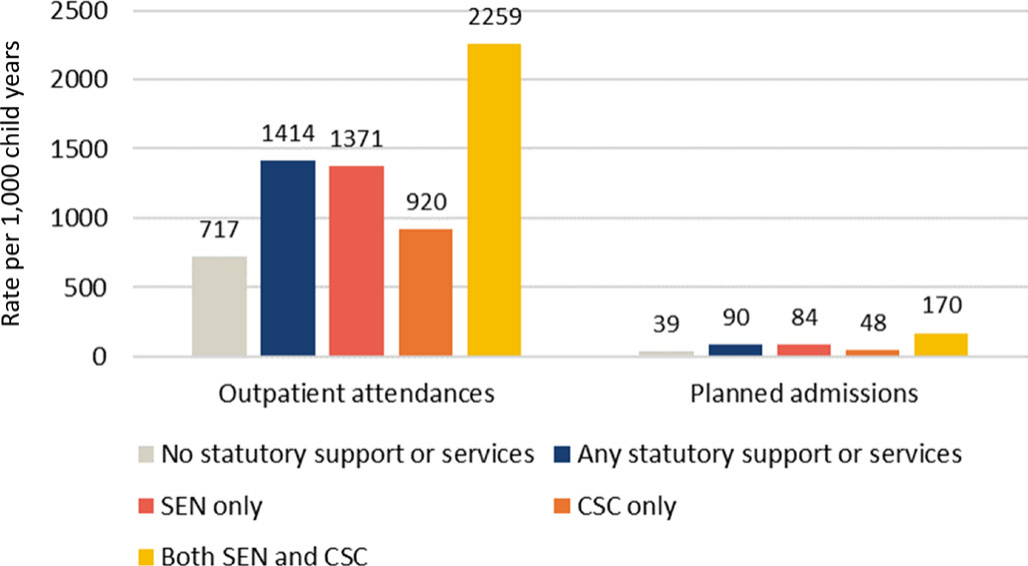
Average rate of planned hospital care per 1000 child-years among secondary school pupils and their peers from 23 March to 31 December (2015–2019), by type of statutory support or service. CSC, children’s social care services; SEN, special educational needs.

Prepandemic, vulnerable adolescents receiving statutory support/services were more likely to use planned hospital care than their peers. For example, in 2018 (the most recent full calendar year for which SEN support and CSC services information is available in the ECHILD database), 34.9% of vulnerable adolescents attended an outpatient appointment compared with 22.6% of their peers (p<0.001; online supplemental table 1). Similarly, in 2018, 4.6% of pupils receiving statutory support/services had a planned hospital admission compared with 2.6% of their peers (p<0.001; online supplemental table 1). Vulnerable adolescents also had higher prepandemic rates of planned hospital care than their peers (figure 1).

### Decreases in planned hospital care during the first 9 months of the pandemic

**Figure 2:**
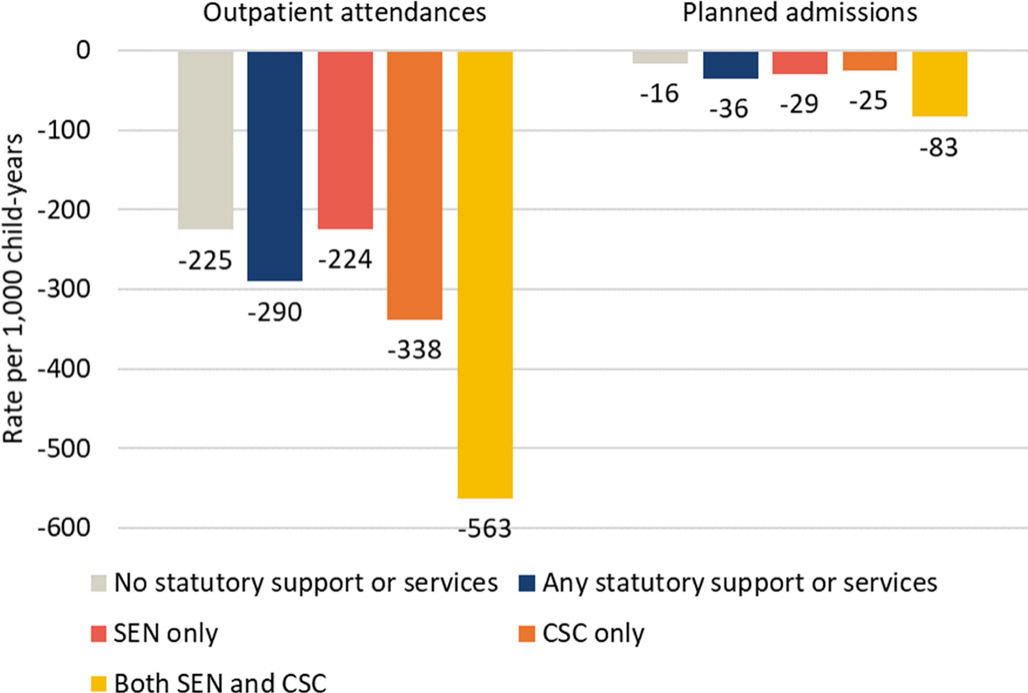
Difference in predicted versus observed rate of planned hospital care per 1000 child-years among secondary school pupils and their peers from 23 March to 31 December 2020, by type of statutory support or service. CSC, children’s social care services; SEN, special educational needs.

From 23 March to 31 December 2020, the rate of outpatient attendances and planned hospital admissions among secondary school pupils was lower than expected (27.5% and 40.1%, respectively; table 2). Larger decreases in rates of planned care were observed for adolescents receiving SEN support or CSC services compared with their peers, with the greatest decrease among those receiving both (figure 2). During the study period, there were 555 012 fewer outpatient attendances than expected among all secondary school pupils and 46 524 fewer planned hospital admissions (table 2). These decreases disproportionately affected vulnerable adolescents. The 21% of adolescents who were receiving statutory support/services accounted for 25% of the decrease in outpatient attendances (138 258 of 555 012) and 37% of the decrease in planned hospital admissions (17 012 of 46 524).

**Table 2:** Difference in predicted and observed rates of planned hospital care from 23 March to 31 December 2020 among pupils in school years 7–11, by type of statutory support or service

	Children (n)	Instances of planned hospital care (n)				Rate per 1000 child-years			
		Predicted	Observed	Deficit	% change (95% CI)	Predicted	Observed	Difference*	% change (95% CI)
**Outpatient attendances**									
Overall	513 683	2 014 154	1 459 142	−555 012	−27.5 (−27.4 to −27.7)	864	626	−238	−27.5 (−27.4 to −27.7)
No support/services	352 958	1 345 303	928 549	−416 754	−31.0 (−30.8 to −31.1)	726	501	−225	−31.0 (−30.8 to −31.1)
Any support/services	160 725	668 851	530 593	−138 258	−20.7 (−20.4 to −20.9)	1400	1110	−290	−20.7 (−20.4 to −20.9)
SEN only	114 244	440 318	366 207	−74 111	−16.8 (−16.5 to −17.1)	1334	1110	−224	−16.8 (−16.5 to −17.1)
CSC only	17 915	79 370	50 674	−28 696	−36.2 (−35.5 to −36.9)	935	597	−338	−36.2 (−35.5 to −36.9)
Both SEN and CSC	28 566	149 163	113 712	−35 451	−23.8 (−23.3 to −24.3)	2371	1808	−563	−23.7 (−23.2 to −24.2)
Planned admissions									
Overall	36 617	115 895	69 371	−46 524	−40.1 (−39.6 to −40.7)	50	30	−20	−40.1 (−39.6 to −40.7)
No support/services	24 294	73 379	43 867	−29 512	−40.2 (−39.5 to −40.9)	40	24	−16	−40.4 (−39.7 to −41.1)
Any support/services	12 323	42 516	25 504	−17 012	−40.0 (−39.1 to −41.0)	89	53	−36	−40.5 (−39.6 to −41.5)
SEN only	8357	27 057	17 410	−9647	−35.7 (−34.5 to −36.9)	82	53	−29	−35.4 (−34.2 to −36.6)
CSC only	1195	4185	2060	−2125	−50.8 (−47.8 to −53.9)	49	24	−25	−50.7 (−47.7 to −53.8)
Both SEN and CSC	2771	11 274	6034	−5240	−46.5 (−44.6 to −48.3)	179	96	−83	−46.3 (−44.4 to −48.1)

Predicted rates were based on Poisson models estimating the number of outpatient attendances/planned admissions that would have occurred in 2020 if the pandemic had not happened, stratified by type of statutory support or service. These models included a linear effect of time (year) to account for ongoing time trends between 2015 and 2019 and a robust sandwich variance estimator. No other covariates were included in the models.

*This column highlights the primary outcome of the analysis: the absolute differences between predicted and observed rates according to vulnerability status, as presented in figure 2.

CSC, children’s social care services; SEN, special educational needs support.

### Mode of outpatient attendances during the pandemic

During the pandemic, 26% of outpatient attendances by adolescents were tele/virtual (453 930 of 1 771 889), compared with just 3% in 2019 (99 478 of 3 410 742). Vulnerable adolescents were less likely than their peers to have an inperson outpatient appointment scheduled (online supplemental table 2) and less likely to attend a scheduled inperson appointment (online supplemental table 3). Overall, this means that during the pandemic a greater proportion of outpatient care was tele/virtual for adolescents receiving statutory support/services compared with their peers (27.8% vs 24.4%, p<0.001; table 3). In absolute terms, this small percentage point difference in the mode of outpatient attendances equates to vulnerable adolescents having 21 641 fewer appointments inperson relative to their peers.

**Table 3:** Type of outpatient attendances among adolescents in school years 7–11 from 23 March to 31 December 2020, by type of statutory support or service

	Total (n)	Inperson		Tele/virtual	
		n	%	n	%
No support/services	1 135 391	858 259	75.6	277 132	24.4
Any support/services	636 498	459 700	**72.2**	176 798	**27.8**
SEN only	440 910	318 326	**72.2**	122 584	**27.8**
CSC only	60 457	45 023	**74.5**	15 434	**25.5**
Both SEN and CSC	135 131	96 351	**71.3**	38 780	**28.7**

Bold indicates a statistically significant difference from ‘no support/services’ reference group at p<0.05.

CSC, children’s social care services; SEN, special educational needs support.

## Discussion

This population-based cohort study of all secondary school pupils in England highlights the large decreases in planned hospital care experienced by adolescents during the initial phase of the COVID-19 pandemic. It illustrates that vulnerable adolescents receiving statutory services/support were disproportionately affected by these decreases and were also less likely to have face-to-face outpatient care than their peers. These disproportionate changes to planned hospital care for vulnerable adolescents are likely to have contributed to further widening of the inequalities^15^ that already existed before the pandemic, in terms of health, education and social care outcomes.^7 8^

This study focused on young people aged 11–16 years whose vulnerabilities could be readily defined from administrative education and social care data and only describes decreases during the first 9 months of the pandemic. The true extent of the decreases in planned hospital care that occurred among all vulnerable children and young people throughout the course of the pandemic will be much greater than our estimates. A further limitation is that, because CSC data are currently only available up to 2018/2019 in the ECHILD database, some young people were misclassified as receiving CSC services during the pandemic when they were not (these individuals might be thought of as having a history of vulnerability) and others as not receiving services when they were. Most children receiving CSC services in 2018/2019 are likely to have been correctly classified as they would also have been receiving services in 2019/2020; approximately half of children who were looked after (52.3%) or in need (48.5%) in 2019/2020 had been for 1 year or more.^16^ Therefore, the overall effect of this misclassification is likely to be an underestimation of the rates of planned hospital care for children receiving CSC services and the decreases they experienced during the pandemic. As more recent data become available in the ECHILD database, it will be possible to update this analysis.

A strength of our analysis is that, in contrast to other studies that have examined decreases in hospital care during the initial phase of the pandemic by only comparing 2020 with 2019 activity levels,^3^ we adopted a modelling approach that accounted for underlying time trends in the previous 5 years. Therefore, our estimates of the decreases in planned care are likely to be more accurate given that the number of hospital admissions and outpatient attendances for children in England is known to be increasing over time.^17 18^ Furthermore, the ECHILD database is a whole population data source that includes all children who had contact with hospitals in England, thereby minimising selection bias relative to other data sources, such as surveys or cohort studies.^5^

Our findings quantify the large decreases in planned hospital care that young people experienced during the initial phase of the pandemic. Although not all planned care may improve outcomes for young people, at least some of these decreases will represent unmet health needs. Adolescence is a period of rapid development when delays to treatment may have long-lasting impact on health and well-being. Planned care that was forgone or deferred could delay diagnoses or treatments, thereby increasing the likelihood of prolonged suffering and complications.^19^ Studies involving adults have shown the physical harms of delays to planned hospital care (such as cancer treatment^20^), as well as the adverse effects on mental health and well-being caused by disruptions and delays to planned hospital care during the pandemic.^21^ Services and practitioners will need to consider how to mitigate the effects of potential unmet needs that may arise in the future from decreases in planned hospital care during the pandemic, including the adverse impact on young people’s mental health and well-being. More research about how delays to planned care for childhood conditions impact outcomes is also urgently needed as few such studies have been conducted.

Some decreases in planned care observed during the initial phase of the pandemic will have been due to changes in young people’s health-seeking behaviour; for example, research involving adults with chronic health conditions highlighted a reluctance to attend hospital for even potentially serious symptoms due to fear of contracting COVID-19.^21^ Services and practitioners will need to encourage young people (and their families and carers) to re-engage with health services to ensure they receive the care they need. This may include working with CSC services, schools and other services that support them. Greater multidisciplinary professional working is one benefit of the shift to remote meetings during the pandemic; for example, doctors were more likely to join virtual Multi-Agency Safeguarding Hub meetings that would had been too difficult to attend inperson prepandemic.^22^ Sustaining the practice of virtual or hybrid multidisciplinary meetings postpandemic may help to reap the benefits of wider engagement and closer working between health and other professionals that support vulnerable young people.

Without the increased use of tele/virtual outpatient appointments, the observed decreases in outpatient attendances during the pandemic would have undoubtedly been much greater. For patients, the benefits of remote consultations during the pandemic include continuity of care when face-to-face contact was not possible and reduced stress because it was not necessary to attend hospital.^23^ However, previous research has found virtual consultations are effective for only a small fraction of patients who are considered ‘suitable’ for this type of care by clinicians,^24^ and the effectiveness for adolescents, particularly those with SEN or receiving CSC services, is unclear. For example, virtual consultations may be more difficult for young people receiving CSC services due to a lack of required resources, such as digital devices and high-speed internet, which was an issue for this group during the pandemic.^22^ Similarly, those receiving SEN support may be more likely to have additional needs, such as learning disabilities, that make meaningful participation in remote consultations more difficult.^25 26^ As well as issues of equity of access, remote consultations for young people also raise concerns in relation to confidentiality and safeguarding, such as health professionals not being able to pick up on non-verbal cues, identify signs of self-harm or know who else is in the room with a patient during a consultation.^22 25^

A key component of the government’s recently published NHS elective recovery plan to reduce hospital waiting lists in the wake of the pandemic is a ‘more personalised’ outpatient model that will reduce standard follow-up care, unless patients request to be seen.^27^ This proposal could further disadvantage vulnerable young people receiving statutory support/services in terms of accessing planned hospital care, given that they were less likely than their peers to attend outpatient appointments and so may be less likely to ‘opt in’ to additional follow-up. As part of the elective care recovery plan, the clinical prioritisation of children and young people on hospital waiting lists is currently being explored in acknowledgement of the potentially profound impact delays to planned care can have on their development.^27^ Our findings suggest that vulnerable children who were affected disproportionately by decreases in planned care during the pandemic may need particular prioritisation given that they already have poorer outcomes than their peers^7 8^ and additionally experienced disruptions to the statutory services that were supporting them. For example, of 509 UK parents surveyed, 20.6% of those whose child had SEN reported receiving no support at all during home schooling, and of those who did receive support 72.5% described it as insufficient.^28^ To be effective, any policy prioritisation for child health in the elective recovery plan will need to be underpinned by additional funding and resources. For example, ring-fenced resources for ‘catch-up’ of NHS care might be further targeted for child health, including the vulnerable groups that have disproportionally missed out on planned hospital care.

Paediatricians and other healthcare practitioners are well aware of the increased morbidity and hence greater use of healthcare among children receiving statutory services/support. However, there is a lack of robust evidence that demonstrates this at a population level, partly due to a lack of administrative data sources that provide a holistic view of children’s lives.^29^ This is the first study to use linked, multidomain administrative data to explore the relationship between healthcare use and education and social care vulnerabilities of all children in England. Our analysis demonstrates that, before the pandemic, young people receiving SEN support or CSC services had greater rates of planned hospital care compared with their peers and that these groups were disproportionately affected by decreases during the initial phase of the pandemic. These findings provide novel, empirical evidence to drive the policy prioritisation of young people receiving statutory services in the future, including any catch-up of planned hospital care postpandemic.

## Data Availability

Data may be obtained from a third party and are not publicly available.

## References

[1] Ladhani SN , Amin-Chowdhury Z , Davies HG , et al . COVID-19 in children: analysis of the first pandemic peak in England. Arch Dis Child 2020;105:1180–5. 10.1136/archdischild-2020-320042 32796006 PMC7431771

[2] Ludvigsson JF . Systematic review of COVID‐19 in children shows milder cases and a better prognosis than adults. Acta Paediatr 2020;109:1088–95. 10.1111/apa.15270 32202343 PMC7228328

[3] Burn S , Propper C , Stoye G . What happened to English NHS hospital activity during the COVID-19 pandemic? 2021. Available: https://ifs.org.uk/uploads/BN328-What-happened-to-English-NHS-hospital-activity-during-the-COVID-19-pandemic.pdf

[4] Maddock J , Parsons S , Di Gessa G . Inequalities in healthcare disruptions during the COVID-19 pandemic: evidence from 12 UK population-based longitudinal studies. medRxiv 2021. 10.1101/2021.06.08.21258546 PMC9561494 36229151

[5] Warner M , Burn S , Stoye G , et al . Socioeconomic deprivation and ethnicity inequalities in disruption to NHS hospital admissions during the COVID-19 pandemic: a national observational study. BMJ Qual Saf 2021. 10.1136/bmjqs-2021-013942. [Epub ahead of print: 25 Nov 2021]. PMC8627367 34824162

[6] Public Health England . No child left behind: understanding and quantifying vulnerability. London, 2020. Available: https://assets.publishing.service.gov.uk/government/uploads/system/uploads/attachment_data/file/913974/Understanding_and_quantifying_vulnerability_in_childhood.pdf

[7] Fleming M , McLay JS , Clark D , et al . Educational and health outcomes of schoolchildren in local authority care in Scotland: a retrospective record linkage study. PLoS Med 2021;18:e1003832. 10.1371/journal.pmed.1003832 34767555 PMC8589203

[8] Fleming M , Fitton CA , Steiner MFC , et al . Using Scotland-wide record linkage to investigate the educational and health outcomes of children treated for chronic medical conditions: a retrospective population cohort study. The Lancet 2019;394:S39. 10.1016/S0140-6736(19)32836-3

[9] Mc Grath-Lone L , Libuy N , Harron K , et al . Data resource profile: the education and child health insights from linked data (ECHILD) database. Int J Epidemiol 2022;51:17. 10.1093/ije/dyab149 34788413 PMC8856003

[10] Green F , Anders J , Henderson M . Who chooses private schooling in Britain and why? 2017. Available: http://www.llakes.ac.uk

[11] Association of Directors of Children’s Services . Elective home education survey 2019, 2019. Available: https://adcs.org.uk/assets/documentation/ADCS_Elective_Home_Education_Survey_Analysis_FINAL.pdf

[12] Jay MA , McGrath-Lone L , Gilbert R . Data resource: the National pupil database (NPD). Int J Popul Data Sci 2019;4:1101. 10.23889/ijpds.v4i1.1101 32935030 PMC7482519

[13] Mc Grath-Lone L , Harron K , Dearden L , et al . Data resource profile: children Looked after return (CLA). Int J Epidemiol 2016;45:716–7. 10.1093/ije/dyw117 27413104 PMC5005948

[14] The Private Healthcare Information Network . The private healthcare information network: annual report 2019-2020, 2020. Available: http://www.phin.org.uk/

[15] Goldfeld S , O'Connor E , Sung V , et al . Potential indirect impacts of the COVID-19 pandemic on children: a narrative review using a community child health lens. Med J Aust 2022;216:364–72. 10.5694/mja2.51368 35066868

[16] Department for Education . Explore education statistics. Available: https://explore-education-statistics.service.gov.uk/data-tables/fast-track/7ce027d2-7cbb-46da-8c60-08d884b70554#subjectTabs-createTable [Accessed 30 Sep 2021].

[17] Ruzangi J , Blair M , Cecil E , et al . Trends in healthcare use in children aged less than 15 years: a population-based cohort study in England from 2007 to 2017. BMJ Open 2020;10:e033761. 10.1136/bmjopen-2019-033761 PMC7228511 32371509

[18] Hargreaves DS , Viner RM . Adolescent inpatient activity 1999-2010: analysis of English Hospital episode statistics data. Arch Dis Child 2014;99:830–3. 10.1136/archdischild-2013-305559 24790134 PMC4145459

[19] Lynn RM , Avis JL , Lenton S , et al . Delayed access to care and late presentations in children during the COVID-19 pandemic: a snapshot survey of 4075 paediatricians in the UK and Ireland. Arch Dis Child 2021;106:e8–2. 10.1136/archdischild-2020-319848 32586927

[20] Hanna TP , King WD , Thibodeau S , et al . Mortality due to cancer treatment delay: systematic review and meta-analysis. BMJ 2020;371:m4087. 10.1136/bmj.m4087 33148535 PMC7610021

[21] Fisher A , Roberts A , McKinlay AR , et al . The impact of the COVID-19 pandemic on mental health and well-being of people living with a long-term physical health condition: a qualitative study. BMC Public Health 2021;21:1–12. 10.1186/s12889-021-11751-3 34620136 PMC8496145

[22] Baginsky M , Manthorpe J . Keeping children and young people safe during a pandemic: testing the robustness of multi-agency child protection and safeguarding arrangements for schools 2020.

[23] Proulx-Cabana S , Segal TY , Gregorowski A , et al . Virtual consultations: young people and their parents' experience. Adolesc Health Med Ther 2021;12:37–43. 10.2147/AHMT.S292977 33953629 PMC8088977

[24] Shaw S , Wherton J , Vijayaraghavan S , et al . Advantages and limitations of virtual online consultations in a NHS acute trust: the vocal mixed-methods study. Health Serv Deliv Res 2018;6:1–136. 10.3310/hsdr06210 29939519

[25] ‘Digital by default’ or digital divide? Virtual healthcare consultations with young people 10-25 years. A joint statement from Young People’s Health Special Interest Group, Adolescent Health Group for Royal College of General Practitioners, Royal College.

[26] Blackburn F , Butler M , Cheung CR . ‘The paediatrician will hear you now’: making virtual outpatient consultations work for children and young people 2018:1041–3. 10.1136/archdischild-2020-321021 33355109

[27] NHS England . Delivery plan for tackling the COVID-19 backlog of elective care, 2022. Available: https://www.england.nhs.uk/coronavirus/wp-content/uploads/sites/52/2022/02/C1466-delivery-plan-for-tackling-the-covid-19-backlog-of-elective-care.pdf 10.1136/bmj.o995 35440437

[28] Thorell LB , Skoglund C , de la Peña AG , et al . Parental experiences of homeschooling during the COVID-19 pandemic: differences between seven European countries and between children with and without mental health conditions. Eur Child Adolesc Psychiatry 2022;31:649–61. 10.1007/s00787-020-01706-1 33415470 PMC7790054

[29] Office for Statistics Regulation . Visibility, vulnerability and voice: the importance of including children and young people in official statistics, 2022. Available: https://osr.statisticsauthority.gov.uk/wp-content/uploads/2022/03/Visibility-Vulnerability-Voice-importance-including-children-young-people-official-statistics.pdf

