# Supplementary material for: Changes in adolescents’ planned hospital care during the COVID-19 pandemic: analysis of linked administrative data
Source: Arch Dis Child. 2022 May 16:archdischild-2021-323616. doi: 10.1136/archdischild-2021-323616 (PMC9157329; doi:10.1136/archdischild-2021-323616)
Supplement: Supplementary file 1 [file archdischild-2021-323616supp001.pdf]

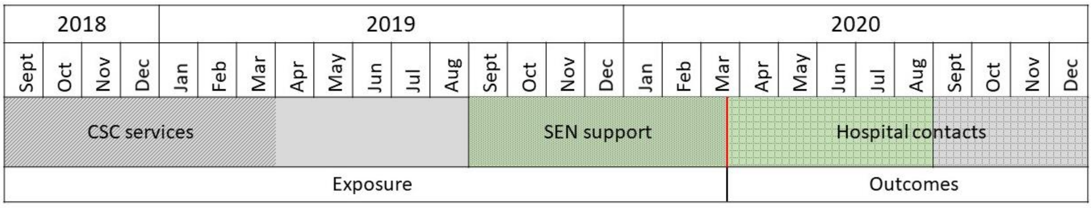

Supplementary Figure 1 Measuring vulnerability (exposure) and hospital contacts (outcomes) for secondary school pupils in years 7 to 11 in 2019/20. SEN = special educational needs; CSC = children's social care. Green shading indicates the 2019/20 academic year. Red line indicates the beginning of the pandemic (23 March 2020). At the time of analysis, the ECHILD Database included data about SEN support up to 31 March 2020 and about CSC services up to 31 March 2019. This means we could identify children who had social care services before the pandemic, but we were not able to identify which children had social care services at the time the pandemic began.

Supplementary Table 1 Prevalence of planned hospital care in 2018 among pupils in school years 7 to 11, by type of statutory support or service.

**Outpatient attendances**	**Number of pupils in years 7 to 11 (N)**	**Number receiving planned hospital care (n)**	**% receiving planned hospital care (n/N)**
No support/services	2,286,790	516,102	22.6%
Any support/services	588,549	205,114	**34.9%**
• SEN only	396,765	140,418	**35.4%**
• CSC only	109,915	27,887	**25.4%**
• Both SEN and CSC	81,869	36,809	**45.0%**
**Planned admissions**			
No support/services	2,286,790	59,458	2.6%
Any support/services	588,549	27,139	**4.6%**
• SEN only	396,765	17,915	**4.5%**
• CSC only	109,915	3,227	**2.9%**
• Both SEN and CSC	81,869	5,997	**7.3%**

SEN = special educational needs support; CSC = children's social care services. Bold indicates a statistically significant difference from "No support/services" reference group at p<0.05. 2018 was the most recent full calendar year for which information related both SEN support and CSC services was available at the time of analysis.

Supplementary Table 2 Type of scheduled outpatient appointments among pupils in school years 7 to 11 from 23 March to 31 December 2020, by type of statutory support or service.

	Total (N)	In-person		Tele/virtual	
		n	%	n	%
No support/services	1,287,546	990,357	76.9%	297,189	23.1%
Any support/services	729,854	540,287	**74.0%**	189,567	**26.0%**
• SEN only	502,107	371,082	**73.9%**	131,025	**26.1%**
• CSC only	71,865	54,890	**76.4%**	16,975	**23.6%**
• Both SEN and CSC	155,882	114,315	**73.3%**	41,567	**26.7%**

SEN = special educational needs support; CSC = children's social care services. Bold indicates a statistically significant difference from the "No support/services" reference group at p<0.05.

Supplementary Table 3 Attendance of scheduled outpatient appointments among pupils in school years 7 to 11 from 23 March to 31 December 2020, by type of statutory support or service and type of appointment.

	All			In-person			Tele/virtual		
	**Attended**	**Scheduled**	**% attended**	**Attended**	**Scheduled**	**% attended**	**Attended**	**Scheduled**	**% attended**
No support/services	1,135,391	1,287,546	88.2%	858,259	990,357	86.7%	277,132	297,189	93.3%
Any support/services	636,498	729,854	**87.2%**	459,700	540,287	**85.1%**	176,798	189,567	93.3%
• SEN only	440,910	502,107	**87.8%**	318,326	371,082	**85.8%**	122,584	131,025	**93.6%**
• CSC only	60,457	71,865	**84.1%**	45,023	54,890	**82.0%**	15,434	16,975	**90.9%**
• Both SEN and CSC	135,131	155,882	**86.7%**	96,351	114,315	**84.3%**	38,780	41,567	93.3%

SEN = special educational needs support; CSC = children's social care services. Bold indicates a statistically significant difference from the "No support/services" reference group at $p<0.05$.
